# Expression and RNAi characterization of neuropeptides underlying developmental regulation in the Colorado potato beetle

**DOI:** 10.3389/finsc.2026.1862257

**Published:** 2026-08-03

**Authors:** Emma E. Terris, Sophie M. Verkoulen, Russell L. Groves, Steven Moen, Sean D. Schoville, Morgan Weissner, Umut Toprak

**Affiliations:** 1 Molecular Ecology Laboratory, Department of Entomology, University of Wisconsin-Madison, Madison, WI, United States; 2 Vegetable Entomology Laboratory, Department of Entomology, University of Wisconsin-Madison, Madison, WI, United States; 3 Department of Statistics, University of Wisconsin-Madison, WI, United States; 4 Molecular Entomology Laboratory, Department of Plant Protection, Ankara University, Ankara, Türkiye

**Keywords:** glycoprotein hormone subunit alpha 2, GPA2, G-protein-coupled receptor (GPCR), *Leptinotarsa decemlineata*, leucine-rich repeat-containing GPCR, LGR1, neuropeptide, RNA interference (RNAi)

## Abstract

Neuropeptides are evolutionarily ancient signaling molecules that act through diverse G protein-coupled receptor (GPCR) pathways to coordinate essential physiological and behavioral processes in insects. Despite their broad functional importance, the specific roles, receptor interactions, and regulatory mechanisms of individual neuropeptides remain underexplored, limiting our understanding of how these signaling systems operate across developmental and physiological contexts. In this study, we characterized transcript expression of five functionally diverse neuropeptides and one GPCR across *Leptinotarsa decemlineata* (Colorado potato beetle) tissues and life stages and subsequently knocked down the glycoprotein hormone subunit alpha 2 (GPA2) and its cognate receptor with glycoprotein hormone subunit beta 5 (GPB5), leucine-rich repeat-containing GPCR 1 (LGR1), for phenotypic investigation. Expression analyses revealed broad and tissue-specific variation among neuropeptides across developmental stages. No significant patterns in mortality, larval weight, or defoliation were detected following dsRNA-mediated knockdown of GPA2 or LGR1 via injection, though in the feeding trial third instar larvae given LGR1 dsRNA weighed significantly more and showed reduced defoliation rates compared the control. State-transition modeling identified significant effects of GPA2 and LGR1 knockdown on developmental timing, specifically during pupal-adult and larval-pupal-adult transitions, respectively, with time in life stage emerging as a key predictor of overall survival. These findings suggest a potential hormonal role for the GPA2-LGR1 signaling system in regulating developmental progression and stabilizing life-stage transitions in *L. decemlineata.*

## 1 Introduction

Neuropeptides are key regulators within the insect endocrine system, produced primarily by neurosecretory cells in the central nervous system (CNS) and by peptide-secreting endocrine tissues throughout the body (1, 2). These CNS-specific peptide hormones are released into the circulatory system or directly onto target tissues within the endocrine system–including the midgut, adipose, muscles, and reproductive organs–where they bind to cell-surface receptors to initiate physiological responses (1, 3–6). Most neuropeptide receptors belong to the G protein-coupled receptor (GPCR) family (7–10), and the pairing between peptides and receptors is often flexible: a single peptide may activate multiple GPCRs, while individual GPCRs may interact with several ligands, sometimes through isoforms with distinct binding preferences (11, 12). This receptor-ligand flexibility allows neuropeptide systems to generate diverse signaling outcomes despite relying on a limited set of intracellular second-messenger pathways (13–16). Together, the organization of the endocrine tissues and the diversity of these signaling interactions underscore the central role of neuropeptides in coordinating critical aspects of insect physiology (10, 17).

Neuropeptides are also considered the most ancient and diverse class of neuromodulators, and they are indispensable to many biological processes, (16–18) encompassing growth and development (molting, diapause), behavior and reproduction (migration, mating, oviposition), metabolism and homeostasis (fat mobilization, protein utilization, blood sugar regulation), water balance, and muscle activity (2, 10, 18–20). Specifically, within invertebrate phyla, more than 50 genes encoding neuropeptides have been identified, and their expression is heterogeneous and finely regulated across tissue types and developmental stages (6, 21–27). Unlike fast synaptic transmission, which occurs in milliseconds, neuropeptide signaling typically acts on slower timescales, ranging from seconds to hours, through intracellular signaling cascades (23, 28–30). This slower and longer-lasting modulation allows neuropeptides to signal changes in physiological state and to influence extended biological processes in response to the environment (18).

These neuromodulators are thought to be evolutionarily conserved, maintaining similar functions across diverse taxa. Consequently, investigating neuropeptide function in invertebrates is of particular interest, as these systems can serve as proxies for understanding more complex signaling pathways in other organisms (6, 10, 17, 20, 31–34). Despite this conservation, the functional roles of many neuropeptides remain unresolved in most non-model insects. Although genomic and transcriptomic surveys have identified numerous neuropeptides and GPCRs, experimental studies verifying their tissue specificity, developmental expression, and physiological effects are still limited. Bridging this gap requires experimental approaches that can more directly link gene expression to phenotype, particularly in species that hold both ecological and agricultural relevance, where understanding endocrine regulation has implications beyond basic physiology.

RNA interference (RNAi) in particular has enabled studies on gene function by reducing target gene expression and, in some cases, creating functional knockouts (35, 36). More recent advances in genetic information, especially through whole-genome sequencing, have further allowed investigation into the neuropeptidome and other signaling systems in insects. Among insects, the Colorado potato beetle (*Leptinotarsa decemlineata*; Say) has become an important model for implementing RNAi-based approaches to investigate gene and neuropeptide function. With extensive genomic and phenotypic research, as well as documented sensitivity to RNAi, *L. decemlineata* is a powerful system for functional gene characterization (36–41). Beyond its value as a physiological model, the agricultural importance of *L. decemlineata* has also positioned it as an important subject of RNAi-based pest management research (42). Development of dsRNA-based bioinsecticides targeting essential genes in *L. decemlineata* has accelerated in recent years, underscoring the need to understand the molecular and endocrine mechanisms that determine RNAi sensitivity, tissue specificity, and downstream physiological effects. This balance between functional relevance and experimental accessibility has already enabled successful RNAi-based studies of neuropeptide function in the species (e.g. 39, 43, 44).

The aim of this study was to investigate the role of uncharacterized neuropeptides in *L. decemlineata*. Candidate neuropeptides were selected based on their documented presence within the genus *Leptinotarsa* and prior annotation in comparative Coleopteran transcriptomic and genomic surveys (6). Targets included glycoprotein hormone subunit alpha-2 (GPA2) with its receptor leucine-rich repeat-containing GPCR-1 (LGR1), Tachykinin, Baratin (NVP-like precursor), Ion Transport Peptide A (ITP-A), and Proctolin. Specific objectives were to (**1**) quantify expression of these five candidate neuropeptides (Proctolin, ITP-A, Baratin, Tachykinin, and GPA2) and the GPCR receptor LGR1 across tissues (midgut, adipose, CNS) and developmental stages (larval, pupal, adult), (**2**) silence neuropeptide GPA2 and its receptor LGR1 using RNAi, and (**3**) evaluate the consequences of gene knockdown for larval growth and survival. Because neuropeptides coordinate numerous physiological processes through signals acting on the CNS, midgut, adipose, and other endocrine tissues, and because many of these peptides shift in expression across life stages, functional characterization in *L. decemlineata* requires examining both their spatial and developmental patterns as well as the consequences of disrupting their signaling. We hypothesized that neuropeptides exhibit stage- and tissue-specific expression patterns consistent with their conserved physiological roles, and that knockdown of neuropeptide GPA2 and its GPCR LGR1 would result in parallel phenotypic outcomes, reflecting their coordinated function within a common signaling pathway. By linking neuropeptide signaling with RNAi-based functional genetics, this study highlights *L. decemlineata* as a model for exploring the physiological roles of neuropeptides, as well as emphasizing that through GPCR-mediated signaling, neuropeptides regulate essential biological processes, making them key candidates for functional analysis.

## 2 Methods

### 2.1 Identification of neuropeptide candidates

Genomic and transcriptomic analyses confirmed the presence of GPA2 (XP_023027110.1) and its receptor LGR1 (XP_023016362.1) in *L. decemlineata*. Tachykinin (XP_023020111.1) was identified with a complete and conserved sequence. Baratin (XP_023017557.1) and ITP-A (XP_023016721.1) were recovered as corrected precursor sequences, while Proctolin (GEEF01088300.1) was found to match the reference form without substitutions or receptor loss in *Leptinotarsa* (6). Collectively, these neuropeptides represent conserved and verifiable signaling systems encompassing ligands and receptors with diverse and potentially functional roles in the *L. decemlineata*.

### 2.2 Leptinotarsa decemlineata dissections

Dissections of *L. decemlineata* were performed on beetles from a laboratory-reared colony maintained in a growth chamber with 24 °C, 70% humidity and a photoperiod of 16:8 L:D cycle. All life stages were fed with non-insecticide treated, fertilized cv. ‘Silverton’ potatoes (*Solanum tuberosum*) reared continuously in a greenhouse. Adult and larval beetles were dissected to isolate the CNS, adipose, and midgut for tissue-specific expression analysis. These tissues were chosen because they represent major centers of neuroendocrine signaling and metabolic regulation in insects (1). Tissues were dissected in PBS under a stereoscopic microscope within 15 minutes in order to prevent significant RNA degradation, using micro-scissors, forceps, and insect pins. Tissues were stored in triplicates (n=10 biological replicates for qPCR) in 0.2 ml microcentrifuge tubes, snap-frozen in liquid nitrogen, and stored at -80 °C. Whole organism samples were also snap-frozen and stored at -80 °C in at least five replicates per life stage, including eggs (frozen in groups of 10), first instar larvae (L1, stored in triplicates), third instar larvae (L3, stored in triplicates), pupae (sexed), and adults (sexed). Life stages were chosen based on developmental milestones, with first and third instars chosen to represent both the small and larger larval stages, while providing some distinction in physiological and morphological traits.

### 2.3 RNA extractions and cDNA synthesis

In order to investigate neuropeptide transcript expression, total RNA was extracted from whole body larvae and dissected tissues in biological triplicates using TRIzol reagent (Invitrogen) and a spin-column based purification with a PureLink RNA Mini Kit (Invitrogen). In the process of purification, RNA was treated with PureLink on column DNase I (Thermo Fisher). All RNA samples were stored at -80 °C and quantified for concentration and purity using a NanoDrop 2000 (Thermo Scientific). Single stranded cDNA for qPCR application was synthesized from total RNA using a SuperScript™ III First-Strand Synthesis kit (Invitrogen). A concentration of 800 ng was input into each reaction. Synthesized cDNA was stored at -20 °C until further downstream analysis.

### 2.4 Quantitative gene expression analysis

Gene expression of the six focal genes (GPA2, LGR1, Tachykinin, Baratin, ITP-A, and Proctolin) was quantified across developmental stages (first instar, third instar, pupae [sexed], and adults [sexed]; all feeding) using quantitative real-time PCR (qPCR). Gene expression data were relativized by the geometric mean expression of three housekeeping genes (RP4, Act-b, and RP18; Primer list for housekeeping genes found in **Supplementary Table 1**) (45, 46) and analyzed using the Pfaffl method (47). Amplification efficiencies were determined from standard curves for each primer pair.

Primers for each neuropeptide (**Supplementary Table 2**) were designed using Integrated DNA Technologies (IDT). Prior to qPCR assays, conventional PCRs were performed for each primer pair to optimize annealing temperature and cycling times. qPCR reactions were run using GoTaq^®^ qPCR Mastermix (Promega) with SYBR^®^ Green I fluorescent dye in a final volume of 10 μL, consisting of 5 μL Mastermix, 0.2 μL of 10 mM forward primer, 0.2 μL of 10 mM reverse primer, 1 μL of cDNA template, and 3.6 μL nuclease-free water. The cycling program included an initial 10 min incubation at 95 °C to activate the polymerase, followed by 40 cycles of denaturation at 95 °C for 10 s, annealing at a primer-specific temperature for 15 s, and extension at 72 °C for 20 s. A melt curve was then generated from 60–95 °C in 1 °C increments every 5 s. All qPCR assays were performed in 0.2 μL tubes using a Qiagen Rotor-Gene-Q instrument.

Standard curves were included on each plate at dilutions of 1, 0.5, 0.2, 0.1, 0.01, 0.001, and 0.0001, along with no-template controls (NTCs) (48). Thresholds were automatically calculated, and values were collected for downstream analysis (**Supplementary Tables 3**, **4**). Runs were only included if amplification efficiency fell between 0.9 and 1.1, and NTCs did not cross threshold. For each sample, ΔCt was calculated as (Ct_calibrator – Ct_sample), where the calibrator was the arithmetic mean of Ct values from n = 3 technical replicates within a specific sample. Relative expression was then computed as (1 + E_target)^ΔCt_target^ divided by the geometric mean of (1+E_ref)^ΔCt_ref^ across the three reference genes. Values > 1 indicate higher transcript expression, and lower expression for values < 1.

To validate target gene expression and confirm tissue specificity prior to qPCR analysis, we compared our candidate neuropeptides to publicly available transcriptomic data from the CPB Gene Expression Atlas (41). All neuropeptide and receptor sequences annotated in *Leptinotarsa* by Veenstra (49) were extracted and cross-referenced with expression matrices spanning larval, pupal, and adult stages and corresponding tissues (central nervous system, midgut, adipose, hindgut, and reproductive tissues). Transcripts per million (TPM) values were log-transformed and visualized in a heatmap to compare relative expression profiles across developmental stages and tissue types. Concordant patterns between the atlas data and published beetle neuropeptide identification (6, 41) were used to confirm gene annotation accuracy and to validate tissue-specific expression trends observed in this study (**Supplementary Figure 1**).

### 2.5 Synthesis of double-stranded RNA

Double-stranded RNA (dsRNA) was generated to conduct gene knockdown trials for GPA2 and LGR1 using the MEGAscript RNAi Kit (Thermo Fisher). We additionally generated a control dsRNA (a dsRNA foreign to the genome of *L. decemlineata*), using the control template from the MEGAscript RNAi Kit. T7 promoters were added to the 5’ end of the previously described primers and used to generate PCR products of ~200 bp of the neuropeptide amplicon sequence. These PCR products were checked for specificity by gel electrophoresis and purified using ethanol precipitation. The purified PCR products were then converted to dsRNA through *in vitro* transcription. The purification process involved DNase I and RNase treatments, as well as a spin column-based purification. The dsRNA was then quantified by spectrophotometry using a NanoDrop 2000 (Thermo Scientific) and normalized to working concentrations for downstream injection and feeding assays.

### 2.6 Phenotypic trial

#### 2.6.1 Choice of dsRNA delivery methods

Direct injection of dsRNA into the hemolymph facilitates systemic knockdown and greater control over the amount of dsRNA applied to each subject, whereas the delivery of dsRNA through feeding minimizes injury-related mortality. In initial trials evaluating dsRNA delivery methods and concentrations, injection treatments resulted in high mortality attributed to puncture wounds. Therefore, we emphasize the results focused on feeding assays, although we provide detail on the injection assay methods and results (see **Supplementary Injection Materials and Methods**, Results: **Supplementary Figures 2–4**; **Supplementary Tables 5–7**).

#### 2.6.2 Feeding of dsRNA

Adult *L. decemlineata* were collected from the Hancock Agricultural Research Station, Hancock, WI (HARS) and offspring were raised in the laboratory for the dsRNA feeding trial. Third (L3) and fourth (L4) instar larvae were fed dsRNA administered on 6 mm potato leaf disks. Treatment groups were established for both instars and included: C1 (untreated control), C3 (500 ng/μL control dsRNA), GD1 (1000 ng/μL GPA2 dsRNA, low-dose GPA2), GD2 (3000 ng/μL GPA2 dsRNA, high-dose GPA2), LD1 (1000 ng/μL LGR1 dsRNA, low-dose LGR1), and LD2 (3000 ng/μL LGR1 dsRNA, high-dose LGR1). Doses were raised compared to published injection protocols to account for nuclease activity in the gut that has been documented to decrease RNAi efficiency (50). Each treatment was replicated with 20 larvae per instar, for n = 120 total larvae, all of the same generation. A total volume of 1μl was applied via micropipette to each disk and air-dried before being fed to individual larvae. All larvae in the dsRNA feeding trial were allowed to feed for two hours on individual disks, and if the entire disk was consumed, larvae were transferred to 150 mm petri dishes with dampened filter paper in groups of 10 with fresh, unaffected potato leaves for the duration of the experiment. Gene knockdown for both candidates in both instars was confirmed via qPCR and gel electrophoresis. Relative gene expression levels were calculated using 2 ^ΔΔCt^ relative expression after normalization to the housekeeping gene RP4 (51). Knockdown efficiency was assessed across dsRNA doses (ng/μL) based on treatment method, 0, 500, 1000 for injection, and 1000, 3000 for feeding. Data can be found in **Supplementary Figure 5**.

#### 2.6.3 Phenotypic assessments

The gene knockdown trials were conducted over 28 days to allow development from larval stages through pupation into adulthood. All *L. decemlineata* were maintained in a growth chamber with 24 °C, 70% humidity and a photoperiod of 16 h light and 8 h dark per day. Phenotypic data was collected daily depending on life stage. Across the entire experiment, larvae were scored as alive, dead, or incapacitated, with incapacitated being defined as the inability to right oneself after being flipped dorsally. Life stage and time to development were also documented with each molt. For the experimental trial, larvae were fed dsRNA as both third and fourth instars, respectively, marked as pupating once fully molted atop shallow soil, and adult when pupal casing was molted. Larval weight (g) and defoliation (%) of potato leaves in a petri dish were taken until all larvae in the replicate pupated or died. Once larvae began to show signs of pupation, they were moved into shallow dishes with soil to replicate a more biologically accurate environment. Once pupation was complete, emerged adults were allowed to feed and reproduce within treatment.

### 2.7 Statistical analysis

In order to test the effect of the dsRNA neuropeptide treatments on typical larval development, phenotypic assessments were statistically analyzed separately in R v 4.5.1 using RStudio (52). Visualizations were created using ggplot2 (53).

#### 2.7.1 Larval weight after knockdown

Average larval weight per dish was analyzed with linear mixed models including treatment replicate (CPB_Group) as a random intercept/repeated factor. Models were fit by maximum likelihood to perform likelihood ratio testing (54). Significant differences between treatment groups were determined using Likelihood Ratio Tests (LRTs), where the full model was compared against reduced models excluding the interaction, treatment, or time. All terms were retained, as their removal significantly reduced model fit. For overall model form, alternatives were compared using Bayesian Information Criterion (BIC) and residual diagnostics (55). A generalized additive model (GAM) provided the best fit and was used to visualize the data as average larval weight over time (56).

#### 2.7.2 Percent defoliation after knockdown

Percent defoliation was analyzed using beta regression with a logit link to account for proportional data. Observations were scaled to fall within 0–1 by applying a constant offset to raw percentages [y=(Defoliation+0.001)/100.002​]. The model included *Treatment*, *Assay Day*, their interaction, and the number of surviving larvae (*Alive*) as fixed effects. Model precision (ϕ) was estimated with an identity link. Wald *z* tests were used for inference, and model fit was assessed with pseudo-R², residual diagnostics, and BIC comparisons to alternative linear and polynomial specifications. Predicted values on the response scale were visualized with treatment means ± SE displayed alongside individual dish trajectories (57).

#### 2.7.3 Survival rate after knockdown

Larval survival was analyzed by Kaplan-Meier methods using individual-level records reconstructed from dish counts (58). Time was assay day (0-28). Treatments were coded as ordered factors, using the uninjected control as the reference. We estimated treatment-specific Kaplan-Meier curves and plotted survival with 95% confidence bands and risk tables over all assay days. Differences among treatments were tested by the log-rank test (survdiff) (59). Pairwise log-rank comparisons were performed with false-discovery rate Benjamini-Hochberg and Bonferroni adjustments for multiple testing (60, 61).

#### 2.7.4 Time to development after knockdown

##### 2.7.4.1 State-transition modeling of beetle development

For individual beetle development data, interval (start-stop) data conveyed state (life stage) and the next observed destination state. States were encoded as: 1 = Alive, 2 = Incapacitated, 3 = Pupating, 4 = Adult, 5 = Dead (absorbing). Allowed/observed transitions included 1→2, 1→3, 1→5, 2→5, 3→4, 3→5, 4→5 (transitions with zero events were fit but not interpreted). All time was measured in days over the 28-day assay duration. For each target transition i→j, there was a transition-specific data set that retained intervals spent in the origin state i. A binary event indicator status was set to 1 if the interval ended with the destination j, and 0 otherwise (including alternative states that were modelled).

##### 2.7.4.2 Model details

Hazards starting from 1, which is Alive, used a Cox-proportional hazards model (62) with a Markov assumption. This means that no prior information about previous states is modeled. Hazards that did not start from 1 relaxed this assumption by including the backward time in the current state, B(t) = stop− start (time since entry to i). This is called a semi-Markov assumption (63).

The general transition-specific Cox proportional hazards model is:


$$\lambda_{ij}(t|X,\;B(t))=\lambda_{0,ij}(t)\;exp\{\beta_{ij}^{\left(trt\right)}X+\beta_{ij}^{\left(B\right)}B(t)+\beta_{ij}^{\left(B^{2}\right)}B{\left(t\right)}^{2}\},$$

Here, *X* is Treatment, which is a factor. To accommodate the difference between models starting from state 1 versus ones not starting from state 1, there are additional terms involving B(t). When starting from 1, both of the leading beta coefficients are set to 0. Only for the Pupating to Adult (3→4) transition, a quadratic term *B*^2^ was enabled in the analysis script. All other non-origin-1 transitions set the leading beta coefficient in front of the B(t) term to 0.

Models were fit using the coxph function in R (62, 64, 65).

##### 2.7.4.3 Inference and contrasts

Inference focused on treatment effects within each transition and the dependence of hazards on time in state *B*(*t*). Estimated marginal means and pairwise contrasts for treatment were fitted from Cox models using emmeans on the model’s linear predictor to control for multiple comparisons within a model (66). Each transition was analyzed in its own risk set (beetles “at risk” were those currently in origin state *i*).

## 3 Results

### 3.1 Neuropeptide gene expression

**Figure 1:**
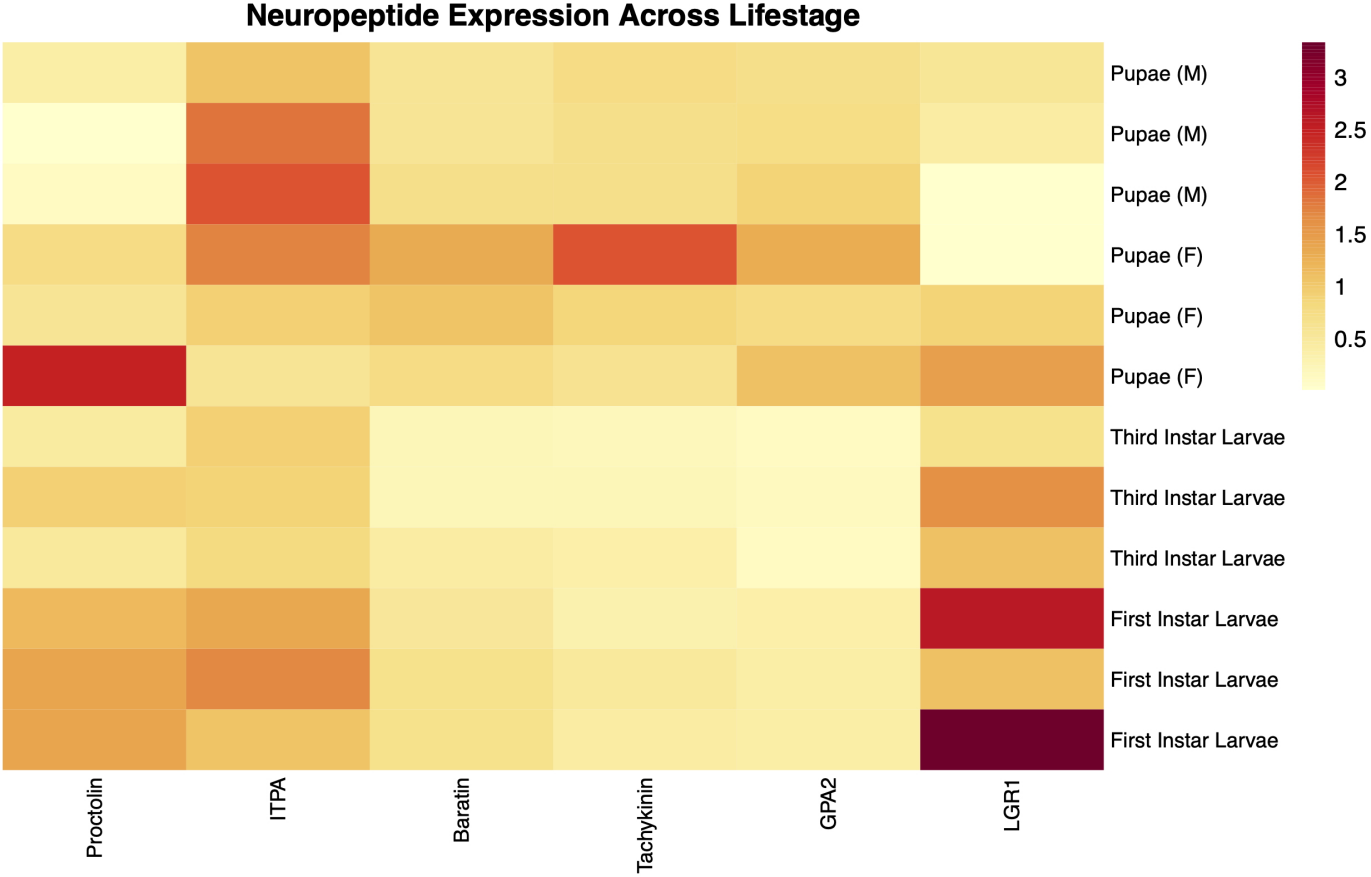
Developmental neuropeptide expression measured by qPCR. Heatmap showing ΔCt values for five neuropeptide genes and one GPCR gene across larval and pupal developmental stages. Rows represent developmental stages (including male pupae, M, and female pupae, F), and columns represent specified genes. Colors indicate expression magnitude, with warmer colors representing higher expression (lower ΔCt) and cooler colors representing lower expression (higher ΔCt), reflected in the scale on the right. All ΔCt values are calculated relative to housekeeping genes.

Across larval and pupal stages, relative neuropeptide expression differed across all six genes (**Figure 1**; **Supplementary Table 3**). Proctolin ranged from extremely low values in pupal males (<0.001) to its highest levels in pupal females (~6.15), with larvae showing intermediate expression (0.15-1.95). ITP-A was broadly detected across stages, spanning 0.35-4.24, and reached its highest values in pupal males, while larval expression remained lower (0.62-1.84). Baratin expression was consistently higher in pupae (0.59-1.74) than in larvae (0.05-0.44). Tachykinin was lowest during larval development (0.03-0.25) and increased in pupae, reaching up to 4.22 in females. GPA2 showed its lowest expression in larvae (0.017-0.16) and higher values across pupal stages (0.49-1.66). LGR1 exhibited the broadest range (0.00017-11.13), with very low expression in pupae, moderate levels in third-instar larvae, and the highest expression occurring in first-instar larvae (up to 11.13).

**Figure 2:**
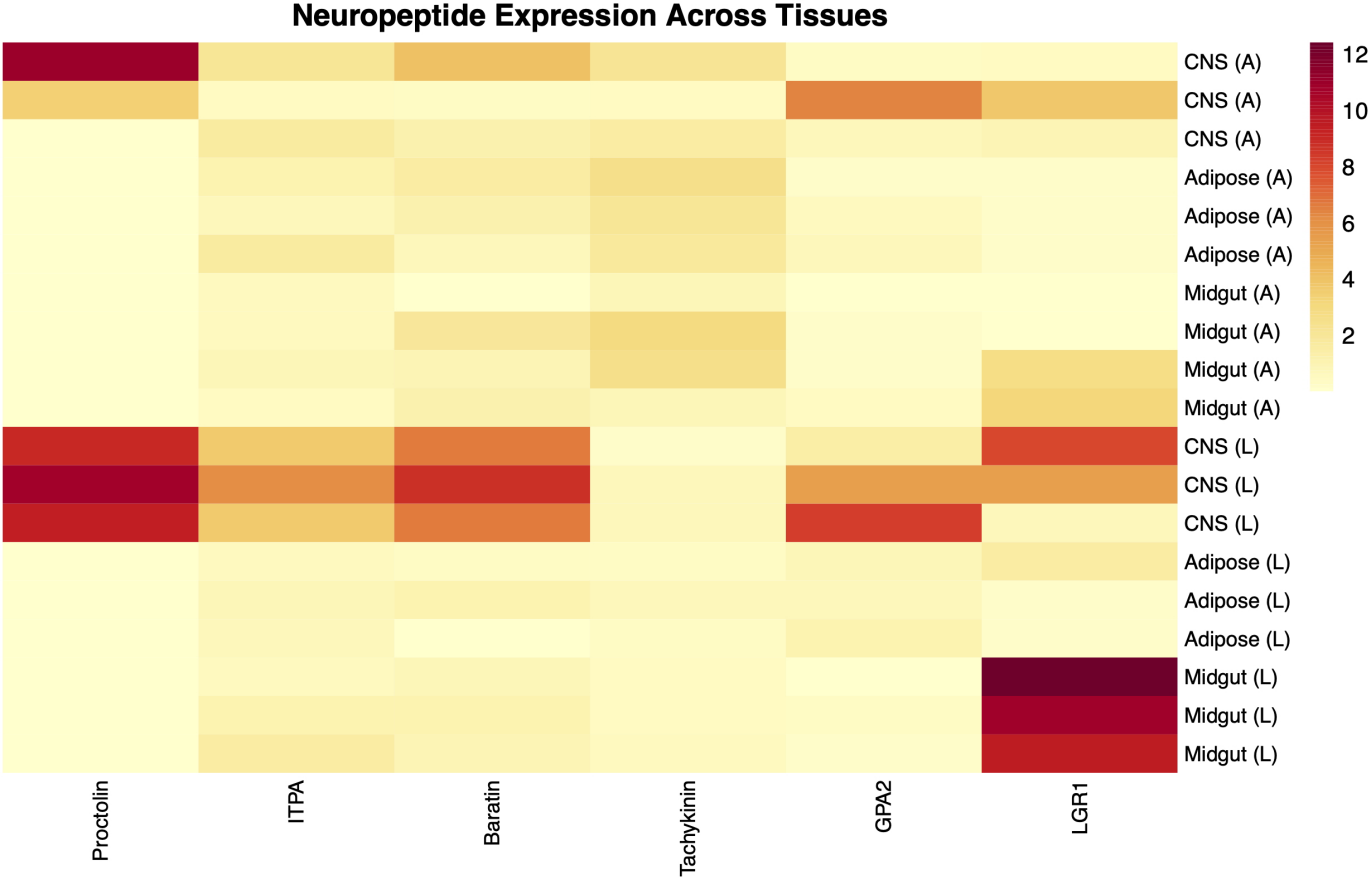
Tissue-specific neuropeptide expression measured by qPCR. Heatmap showing ΔCt values for five neuropeptide genes and a GPCR across larval and adult tissues. Rows represent sampled tissues (central nervous system (CNS), midgut, and adipose labeled L for larval and A for adult), and columns represent specified genes. Colors reflect expression magnitude, with warmer colors indicating higher expression (lower ΔCt) and cooler colors indicating lower expression (higher ΔCt), reflected on the scale on the left. All ΔCt values are calculated relative to housekeeping genes.

Tissue-specific patterns were also diverse across the neuropeptides (**Figure 2**; **Supplementary Table 4**). Proctolin was most highly expressed in CNS tissue in both larvae and adults (0.002-119.5), and nearly absent from adipose and midgut (<0.02). ITP-A expression was also highest in CNS (adult: 0.19-4.42; larval: 13.1-37.1) and lower in adipose (0.26-1.05) and midgut (0.25-2.46). Baratin expression ranged from 0.0003-77.3, with its strongest signal in larval CNS (43.7-77.3) and much lower values in adult adipose and midgut. Tachykinin showed the opposite pattern: low in larval CNS (0.027-0.48) but elevated in adult adipose and midgut (3.47-8.86). GPA2 expression was dominated by CNS tissue (larval: 1.94-67.97; adult: 0.12-41.16), with low values in adipose and midgut (<1.2). LGR1 was most strongly expressed in midgut tissue, particularly in larvae (89.2-154.1), with moderate CNS expression and the lowest levels in adipose tissue.

### 3.2 Larval weight after knockdown

**Figure 3:**
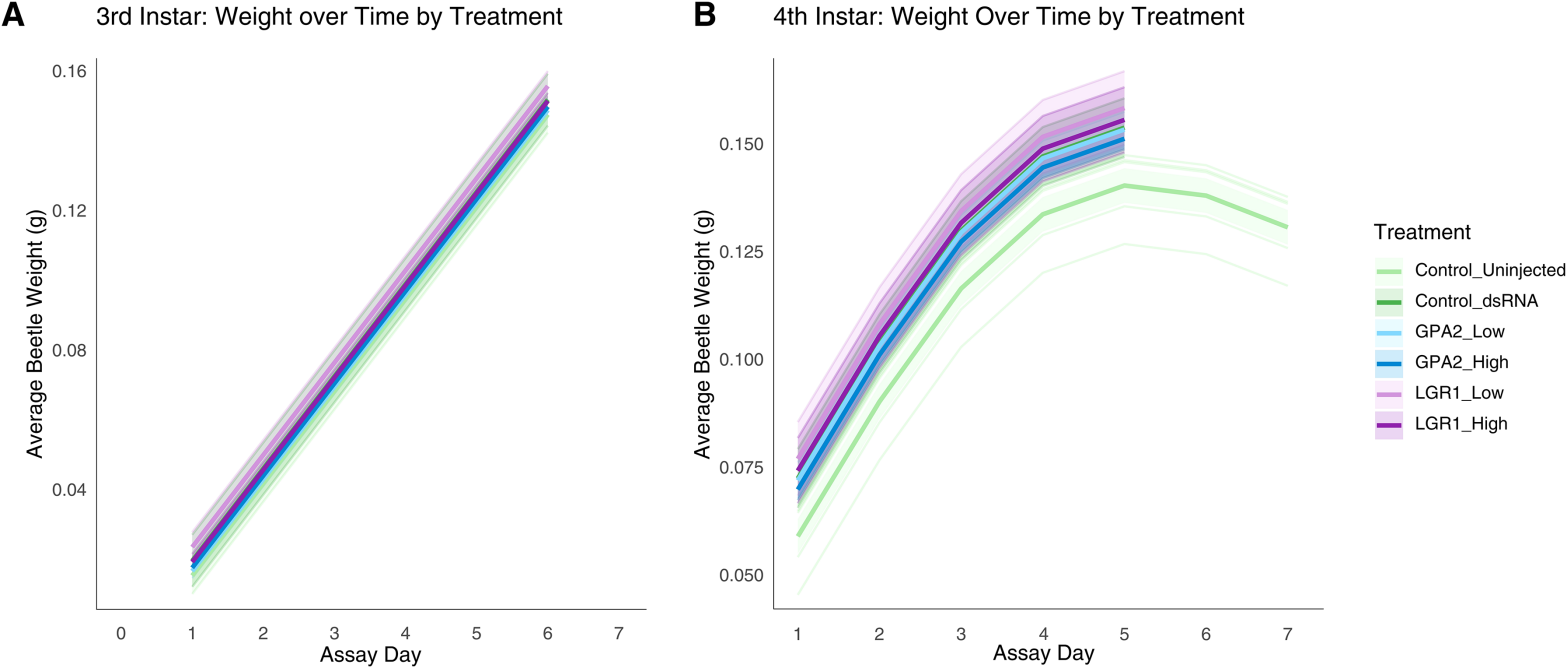
Larval weight by treatment over time. **(A)** Average third instar larval weight (g) across larval feeding assay days (1–6) for all treatments: uninjected control (Control_Uninjected), dsRNA-injected control (Control_dsRNA), and dsRNA treatments targeting GPA2 and LGR1 at low and high concentrations (GPA2_Low, GPA2_High, LGR1_Low, LGR1_High). Lines represent predicted fits from a linear model, and shaded ribbons indicate 95% confidence intervals. **(B)** Average fourth instar larval weight (g) across larval feeding assay days (1–7) for the same treatments. Lines represent predicted fits from a generalized additive model (GAM), and shaded ribbons indicate 95% confidence intervals.

Average beetle weight in the third instar feeding trial increased significantly over the entire assay (+0.0231 g/day, *p* < 0.001). No treatment differed from the uninjected control on day 0 of the assay. Compared to the control, both low-dose LGR1 and high-dose LGR1 treatments showed significantly greater rates of weight gain (+0.0049 g/day, *p* = 0.032; +0.0053 g/day, *p* = 0.020). Control dsRNA and both low- and high-dose GPA2 treatments did not differ significantly from the control (**Figure 3A**).

Average beetle weight in the fourth instar feeding trial also increased significantly and steadily across the assay (+0.0147 g/day, *p* < 0.001). On day 0, no treatment group differed significantly from the control. Growth trajectories across treatments were generally similar, and no treatment × day interaction terms were significant (*p* = 0.91). The uninjected control treatment did feed for longer than all other treatments, but this did not significantly affect the model. Overall, there was no evidence for treatment-specific differences in weight (**Figure 3B**).

### 3.3 Rate of defoliation after knockdown

**Figure 4:**
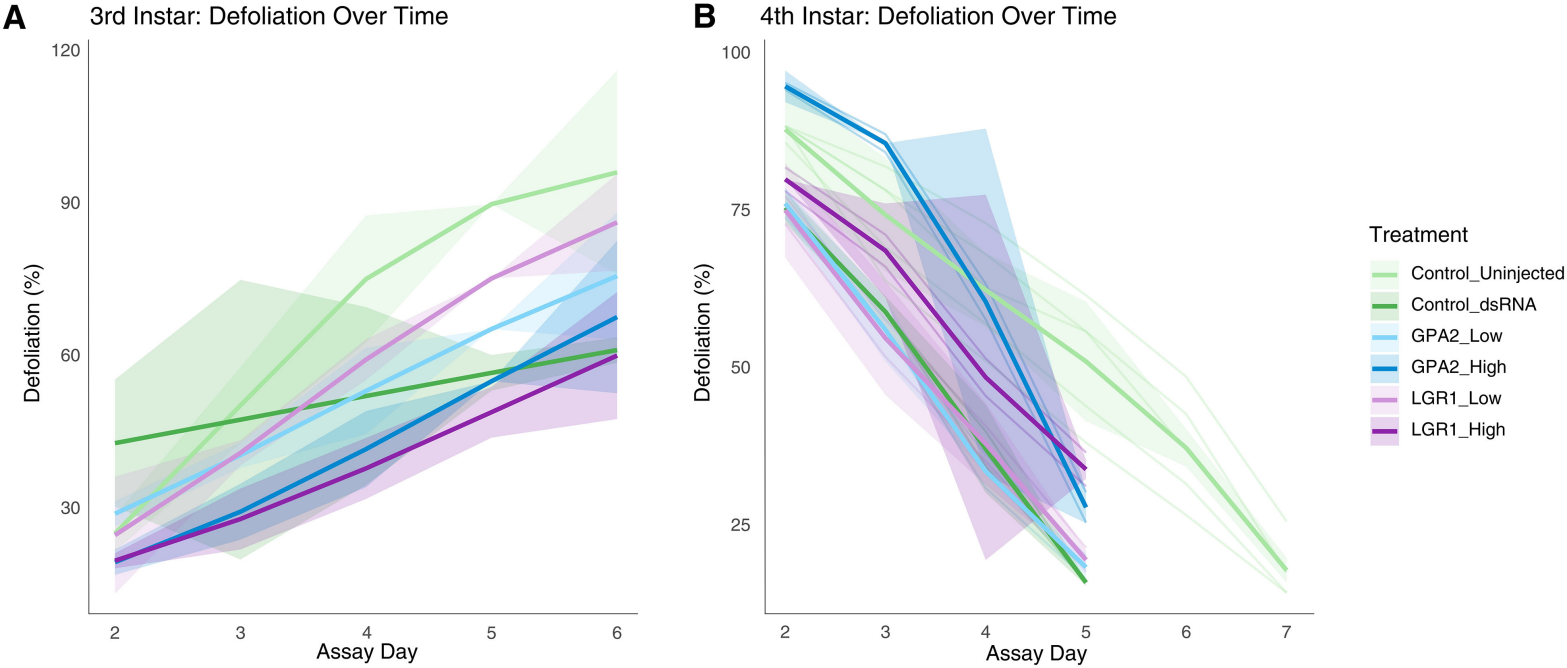
Larval defoliation by treatment over time. **(A)** Defoliation by third instar larvae across larval feeding assay days (2–6) by treatment, measured as the percentage of the potato leaf defoliated, for all treatments: uninjected control (Control_Uninjected), dsRNA-injected control (Control_dsRNA), and dsRNA treatments targeting GPA2 and LGR1 at low and high concentrations (GPA2_Low, GPA2_High, LGR1_Low, LGR1_High). Lines represent predicted values from beta regression, and shaded ribbons denote standard errors. **(B)** Defoliation by fourth instar larvae across larval feeding assay days (2–7) by treatment, measured the same way and for the same treatments. Lines represent predicted values from beta regression, and shaded ribbons denote standard errors.

For the rate of defoliation (%) in the third instar feeding trial, a beta regression with a logit link was applied (pseudo-R² = 0.38; ϕ = 3.33, *p* < 0.001). Defoliation increased significantly over days (*p* < 0.001). A significantly reduced rate of defoliation was observed when compared to the control was seen in dsRNA control treated larvae (*p* = 0.007), and high-dose LGR1 treated larvae (*p* = 0.038). Low- and high-dose GPA2 treatment groups showed marginal reductions in feeding (*p* = 0.06 and 0.07, respectively), while low-dose LGR1 did not differ from the control (**Figure 4A**).

Defoliation across assay days in the fourth instar feeding trial was similarly analyzed using beta regression with a logit link (pseudo-R² = 0.393; ϕ = 1.34, *p* < 0.001). Baseline defoliation at assay day 0 did not differ significantly across treatments (all *p* > 0.10). Overall defoliation decreased significantly over time (*p* < 0.001). No treatment × day interactions were significant, indicating that the rate of decline in defoliation was consistent across treatments. The number of beetles alive on each day showed a weak positive trend (*p* = 0.097), suggesting that higher beetle survival could have contributed to greater cumulative defoliation, though this effect was not significant. All treatments and controls had a decreasing defoliation rate as the assay progressed (**Figure 4B**).

### 3.4 Survival after knockdown

**Figure 5:**
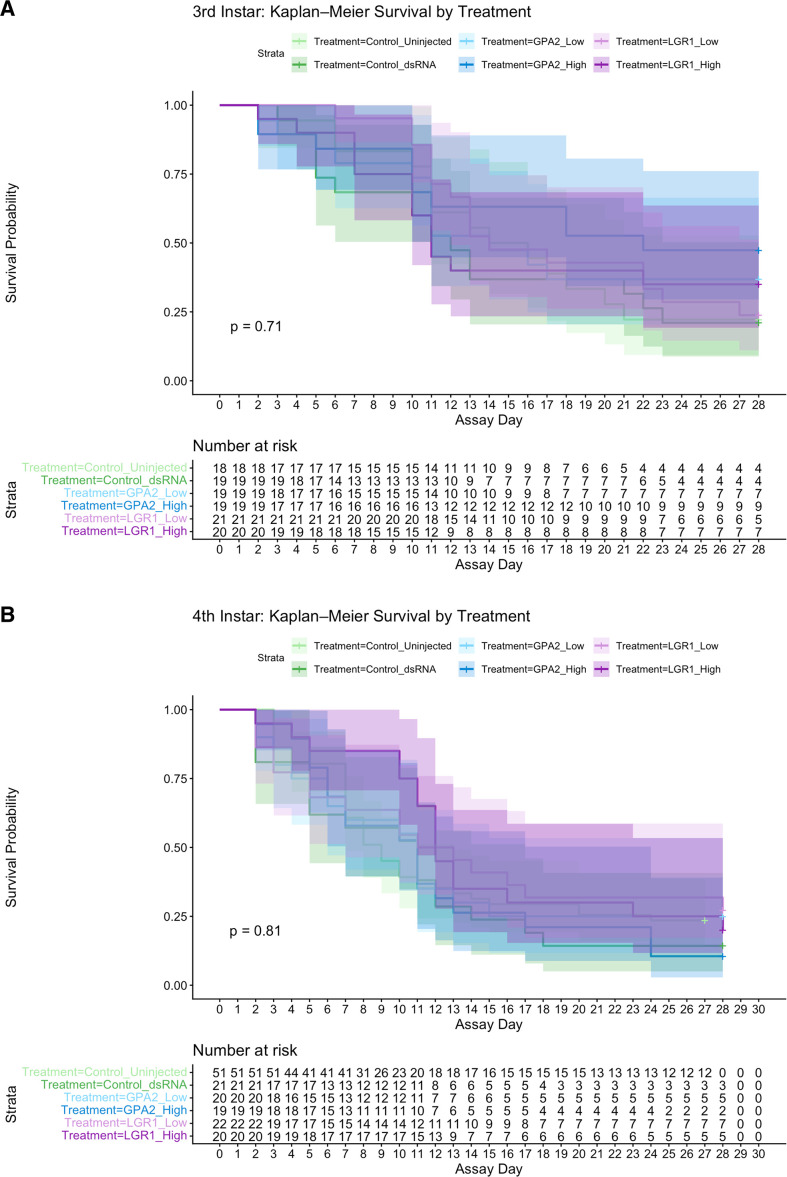
Larval survival by treatment over time. **(A)** Kaplan–Meier survival probability of third instar larvae across assay days (0–28) by treatment: uninjected control (Control_Uninjected), dsRNA-injected control (Control_dsRNA), and dsRNA treatments targeting GPA2 and LGR1 at low and high concentrations (GPA2_Low, GPA2_High, LGR1_Low, LGR1_High). Shaded areas represent 95% confidence intervals, the number-at-risk table below indicates the individuals at risk at each time point, and tick marks (+) denote censored observations. Survival did not differ significantly among treatments (log-rank p = 0.71). **(B)** Kaplan–Meier survival probability of fourth instar larvae across assay days (0–30) for the same treatments. Shaded areas represent 95% confidence intervals, the number-at-risk table below indicates the individuals at risk at each time point, and tick marks (+) denote censored observations. Survival did not differ significantly among treatments (log-rank p = 0.81).

Kaplan-Meier survival curves for the third instar feeding trial showed similar survival dynamics across treatments over the 28-day assay (**Figure 5A**). The overall log-rank test detected no significant differences among treatments (χ² = 2.9, df = 5, *p* = 0.71). Pairwise log-rank comparisons revealed no significant differences between treatments, with all adjusted p-values exceeding 0.85 after the Benjamini-Hochberg correction and equal to 1.0 after the Bonferroni correction.

Kaplan-Meier curves for the fourth instar feeding trial showed comparable survival trajectories to the third instar group across all treatments over the 28-day assay (**Figure 5B**). The overall log-rank test detected no differences in survival among treatment groups (χ² = 2.3, df = 5, *p* = 0.80). Pairwise log-rank comparisons revealed no significant differences between treatments after multiple-test correction (all adjusted *p* ≥ 0.81). Overall, there were no significant differences in survival between any of the treated fourth instar groups and the control groups.

### 3.5 Development After Knockdown

#### 3.5.1 Third instar treatment

Across transitions originating in stage 1, there was no evidence that treatments altered either progression or incapacitation risk. The larval to incapacitated transition (1→2) did not occur in any individual (n = 120, events = 0), so the model could not estimate treatment-specific hazards, or developmental stage transition differences due to treatment (LRT = 0 on 0 df, *p* = 1.0). For the larval to pupal transition (1→3), treatment hazard ratios relative to the control group (3FC1) were all non-significant (all *p* ≥ 0.14), and the overall model was not significant (LRT = 7.50 on 11 df, *p* = 0.76; n = 120, events = 61).

In the life stage transition between pupae and adults (3→4), both treatment and time in the pupal state influenced life stage progression hazards. The semi-Markov specification included a quadratic time-in-state effect: the linear *B*(*t*) term was not significant (coef 1.422, HR per unit *B*(*t*) = 4.15, *p* = 0.121), but the squared term was ( $I(Bt^{2})$); coef −0.206, HR = 0.814, *p* = 0.025), indicating a peak in hazard at non-intermediate pupal durations. Relative to the control treatment (FC1), the dsRNA control (FC3) showed a lower hazard of pupal-adult transition (coef −3.030, HR = 0.048, *p* = 0.0117), and high-dose LGR1 (FLD2) also reduced the hazard (coef −3.775, HR = 0.023, *p* = 0.0148). High-dose GPA2 (FGD2) showed a similar tendency (coef −2.030, HR = 0.13, *p* = 0.061), while other treatments did not differ from the control (all *p* > 0.14). The overall model fit was significant (LRT = 71.35 on 13 df, *p* = 5.0 × 10^−10^; n = 61, events = 41), indicating duration-dependent pupal development with reduced 3→4 transition under some dsRNA treatments.

For the life stage transition between pupae and death (3→5), the semi-Markov model detected an effect of time in the pupal state and rare treatment-dependent jump events. The *B*(*t*) term was negative (coef -1.469, HR per unit *B*(*t*) = 0.230, p = 9.76 × 10^−5^), indicating that direct pupal death occurred mainly soon after entering the pupal state and that the 3→5 hazard declined with increasing pupal duration. Relative to the control (3C1), several treatments showed reduced estimated hazards of pupal death, including dsRNA control (3C3; coef −4.471, HR = 0.0114, p = 0.0174), low-dose GPA2 (FGD1; coef −4.183, HR = 0.0153, p = 0.0179), and low-dose LGR1 (FLD1; coef −4.157, HR = 0.0156, p = 0.0154). Other treatments had no observed 3→5 events, producing quasi-separation and large standard errors with coefficients tending toward −∞ and p ≈ 1.0. The overall model was significant (LRT = 36.48 on 12 df, p = 3.0 × 10^−4^; n = 61, events = 13), so direct pupal death was rare, concentrated early after pupation, and absent or greatly reduced in several dsRNA treatments.

Analysis of the life stage transition between adults and death (4→5) showed an effect of time spent in the adult stage but no detectable differences among treatments. The semi-Markov time-in-state term was negative (*B*(*t*); coef −0.924, HR per unit *B*(*t*) = 0.397, p = 2.22 × 10^−4^), indicating higher death hazard soon after adult emergence and lower hazard with increasing adult duration. None of the treatment contrasts relative to the control (3C1) were statistically significant (n = 41, events = 11). The overall model was significant (LRT = 39.67 on 12 df, p = 8.0 × 10^−5^), driven by the *B*(*t*) effect, indicating that adult survival was primarily a function of time since emergence rather than treatment.

#### 3.5.2 Fourth instar treatment

Again, modeled transitions starting from stage 1, or larvae, did not include a *B*(*t*) term, as there is no preceeding state in this design. The larval to incapacitated transition (1→2) had all hazards as non-significant (all *p* ≥ 0.42), and the overall model was not significant (LRT = 10.19 on 10 df, *p* = 0.4241; n = 120, events = 7). For the transition from larvae to pupae (1→3), there were significant treatment effects on progression. High-dose GPA2 (4FGD11) produced the strongest effect (coef = 1.95, HR = 7.00, *p* < 0.0001), indicating a substantially increased hazard of pupation, or a faster transition from the larval to pupal stage. Low-dose LGR1 (coef = 1.19, HR = 3.29, *p* = 0.014) and high-dose LGR1 (coef = 0.90, HR = 2.47, *p* = 0.047) also significantly elevated pupation hazard. The overall model was significant (LRT = 18.41 on 10 df, *p* = 0.048).

In the life stage transition between pupae and adults (3→4), treatments with no observed emergence events were excluded to allow model convergence. The semi-Markov model with both *B*(*t*) and *B*(*t*)² terms identified clear duration-dependent and treatment-related effects. The linear *B*(*t*) term was positive (coef = 5.473, HR = 238.2, *p* = 0.072), and the squared term was negative (coef = −0.682, HR = 0.506, *p* = 0.070), indicating that emergence hazard peaked at early into pupal duration and declined as individuals remained longer in the pupal state. Relative to the control, most treatments did not significantly alter the progression hazard. However, high-dose GPA2 treatment exhibited an increased hazard (coef = 2.311, HR = 10.09, *p* = 0.059), and low-dose LGR1 treatment showed a significant increase in emergence probability (coef = 10.344, HR = 3.1 × 10^4^, *p* = 0.0077). All other treatment effects were non-significant (*p* ≥ 0.13); however, the overall model was significant (LRT = 54.26 on 11 df, *p* = 0.0010). These results indicate that, under feeding-based dsRNA exposure, pupal development is sensitive to both time in state and specific dsRNA treatments, with certain GPA2 and LGR1 treatments accelerating emergence to adulthood.

For the transition from pupa to death (3→5), the semi-Markov model identified a duration effect of time in the pupal state but no clear treatment differences. The *B*(*t*) term was significantly negative (coef = -0.689, HR per unit *B*(*t*) = 0.502, p = 0.0047), indicating that pupae that died did so soon after entering the pupal stage, and the hazard of direct pupal death decreased with increasing time in state. All treatment coefficients were non-significant (all p ≥ 0.35), although a few groups showed extreme estimates with very wide confidence intervals due to zero events in those treatment cells. The overall model was significant (LRT = 31.14 on 11 df, p = 0.001; n = 61, events = 15), driven primarily by the *B*(*t*) effect rather than treatment.

For the transition from adult to death (4→5), time in the adult state significantly affected mortality, with no consistent treatment effects. The *B*(*t*) term was significantly negative (coef = -0.811, HR per unit *B*(*t*) = 0.444, *p* = 0.0044), indicating that newly emerged adults had the highest death hazard and that risk declined with increasing time since adult emergence. No treatment contrasts were statistically significant (all *p* ≥ 0.15). The overall model was significant (LRT = 34.42 on 10 df, *p* = 0.000156; n = 29, events = 11), again primarily reflecting the time-in-state effect.

#### 3.5.3 Overall dsRNA effects on development

**Figure 6:**
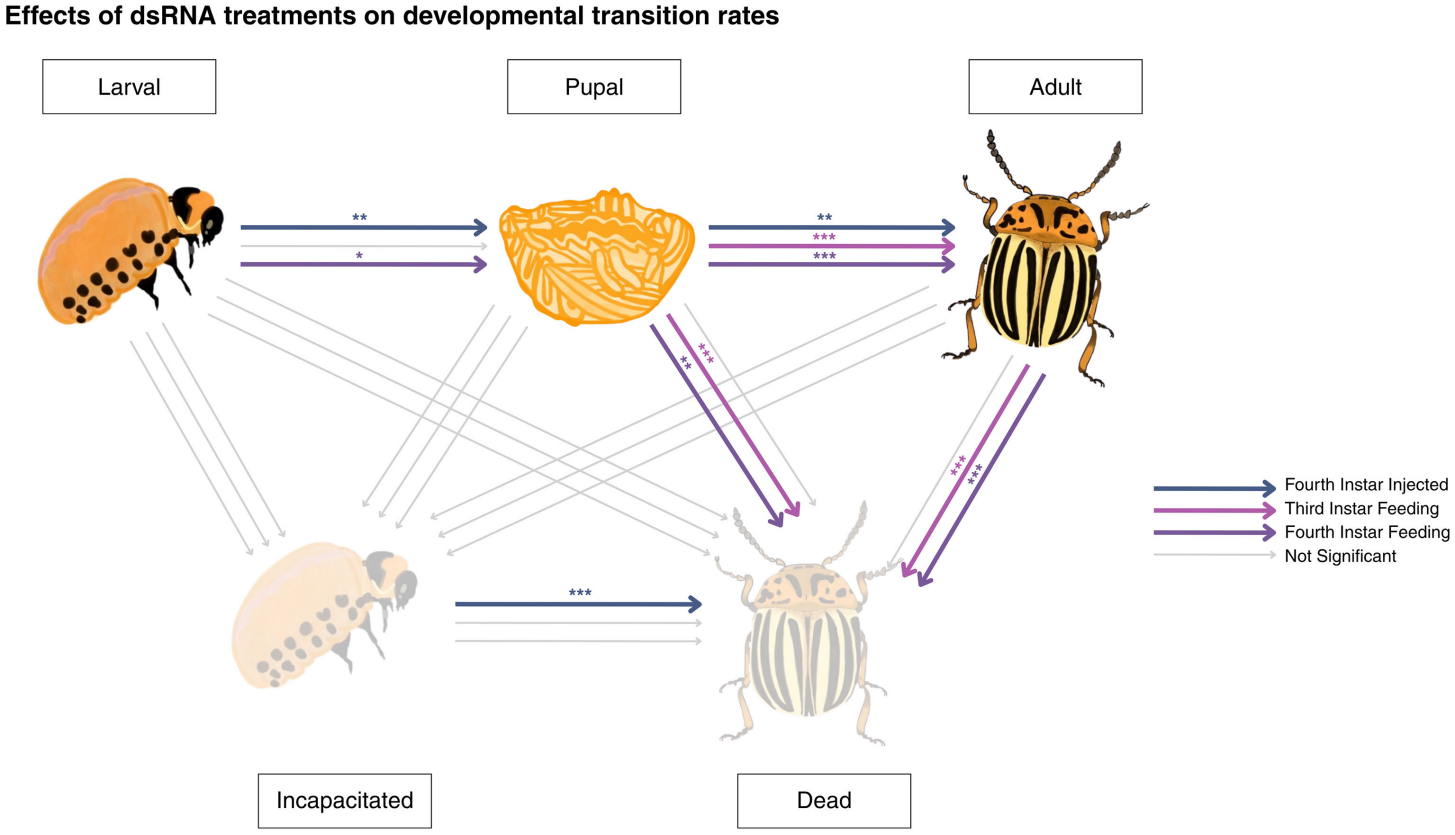
Effects of dsRNA treatments on developmental transition rates. Diagram summarizing significant changes in developmental transition hazards under GPA2 and LGR1 dsRNA exposure across injection and feeding assays. Arrows indicate transitions among larval, pupal, adult, incapacitated, and dead states. Color corresponds to assay type: dark blue = fourth-instar dsRNA injection, light purple = third-instar dsRNA feeding, dark purple = fourth-instar dsRNA feeding, and grey = non-significant transitions. Asterisks denote significance levels from Cox models (*p* < 0.05: *p < 0.01: **p < 0.001: ***).

Across all injection treatments (**Supplementary Injection; Materials and Methods; Results**), baseline mortality was substantially elevated, reflecting methodological injection stress rather than gene-specific effects. Even with this elevated background mortality, several developmental patterns observed in the feeding assays were also present in the injected beetles: GPA2 and LGR1 knockdown consistently altered later stage larval and pupal transition rates across assays, and these effects remained detectable when analyses were restricted to individuals that survived injection. The summary of transition outcomes in **Figure 6** illustrates the same developmental steps showing significant developmental changes under GPA2/LGR1 dsRNA across feeding and injection treatments. Together, these shared patterns strengthen the conclusion that the observed changes in developmental timing arise from disruption of the GPA2/LGR1 pathway rather than from delivery-specific effects.

## 4 Discussion

### 4.1 Overview of key findings

By mapping neuropeptide expression of Proctolin, ITP-A, Baratin, Tachykinin, and GPA2, as well as the neuropeptide receptor LGR1, across tissues and life stages we demonstrate the potential roles of these uncharacterized neuropeptides in *L. decemlineata*. Transcript expression in this study for each neuropeptide aligns with previous research in this beetle, as well as adding previously undocumented neuropeptide expression from CNS tissue. Furthermore, by combining these patterns with RNAi-based functional tests for two of the neuroendocrine targets, this study identifies developmental timing as the major physiological process influenced by GPA2/LGR1 in *L. decemlineata*, while growth and survival remained largely unaffected.

### 4.2 Neuropeptide expression patterns and functional context

The structured expression patterns revealed by qPCR data suggest distinct physiological roles for each neuropeptide. Considering these patterns alongside functional roles described in other insects helps to identify how these neuropeptides may operate within the *L. decemlineata* endocrine system. Importantly, Proctolin was the first neuropeptide to be characterized in arthropods and appears to be absent in Hymenoptera, Lepidoptera, and select Diptera (67). Across multiple arthropod orders, this neuropeptide has been shown to have very diverse functions, including modulation of muscle tissue, factoring release of other hormones, and influencing sexual behavior (20, 67). Proctolin showed elevated expression in the larval and adult CNS and in first-instar larvae. This pattern is consistent with studies in *Rhodnius prolixus*, where Proctolin transcripts and immunoreactive processes occur in the CNS and in peripheral tissues, including salivary glands, reproductive organs, the midgut, hindgut, and heart. Proctolin is biologically active in these tissues, stimulating muscle contraction in the gut and increasing heartbeat frequency (68). Similar findings in *Drosophila melanogaster* and other insects show that Proctolin acts through Proc-R to modulate muscle activity and heart rate and is released from CNS neurons to influence peripheral targets (69). The strong CNS-biased expression observed in *L. decemlineata* aligns with these established roles and suggests comparable neuromuscular regulatory functions.

ITP-A showed its highest expression in the larval CNS and in both male and female pupae. In other insects, ITP-A functions as an inosine triphosphate pyrophosphatase involved in maintaining nucleotide pool integrity, regulating ion and fluid transport and ovarian maturation, as well as regulating the activity of ATP/GTP-binding proteins (20, 70, 71). Although its broader physiological roles remain less well-defined, ITP-related peptides in *D. melanogaster* and other species are expressed in neural tissues and contribute to processes involving ion balance and nucleotide metabolism (72). In *Aedes aegypti*, ITP-like peptides have also been found to modulate excretion, blood feeding and egg laying in females, and reducing egg viability (20, 71). The greater expression in CNS tissue and pupae in *L. decemlineata* is therefore consistent with roles in developmental or metabolic regulation, however this study did not find that ITP-A expression was greater in females than in males.

Expression of Baratin was highest in the larval CNS and moderate in female pupae. In *Tribolium castaneum* and *Leucophaea maderae*, Baratin (or NVP-like peptides) is produced from a larger precursor and is well conserved across Coleoptera (6). Baratin-immunoreactive interneurons are distributed throughout the CNS, with projections into thoracic and abdominal ganglia and neurosecretory cells interacting with the corpora cardiaca. These peptides have been shown to modulate gut activity and motor patterns in abdominal ganglia, including activity in the hindgut and oviduct (73). The CNS-biased expression observed in *L. decemlineata* larvae is consistent with these reported roles in neural and visceral motor regulation, however we had hypothesized higher expression of Baratin in midgut tissue in both adults and larvae.

Tachykinin expression in *L. decemlineata* was low in larval stages and higher in pupae, with elevated levels in adult adipose and midgut. However, this neuropeptide displayed the lowest relative expression across all tissue samples when compared to the other five genes. In *D. melanogaster*, *Locusta migratoria*, and *L. maderae*, Tachykinins act in pathways involved in feeding behavior, locomotion, nociception, aggression, metabolic stress responses, hormone release, and regulation of lipid metabolism in the intestine (20, 27, 74, 75). Tachykinin peptides also serve as neuromodulators in crustacean stomatogastric motor circuits and are expressed widely across arthropod CNS regions (20, 27). Interestingly, evolutionary analyses indicate that Coleoptera have experienced reductions in tachykinin paracopy number, and *L. decemlineata* retains only two tachykinin peptides relative to the eight ancestral domains observed in most beetles (6). Observed qPCR expression patterns suggest that tachykinin activity in this beetle may shift toward endocrine and digestive functions during later development rather than early larval stages.

### 4.3 GPA2/LGR1 expression patterns and functional interpretation

The GPA2/GPB5-LGR1 system in *L. decemlineata* showed separation between neuropeptide and receptor expression: GPA2 expression was higher in larval and adult CNS and across pupal stages, whereas LGR1 expression was concentrated in larval and adult midgut and in early larval instars. Glycoprotein hormones are essential regulators of physiology across vertebrates and invertebrates. In vertebrates, the classical glycoprotein hormones, follicle-stimulating hormone (FSH), luteinizing hormone (LH), thyroid-stimulating hormone (TSH), and Chorionic Gonadotropin (CG) control growth, metabolism, and reproductive events such as follicle maturation and ovulation. Their structural analog in invertebrates, the heterodimeric hormone formed from GPA2 and its counterpart glycoprotein subunit beta-type 5 (GPB5) (thyrostimulin in vertebrates), forms part of an evolutionarily conserved signaling system that acts through the GPCR LGR1 (76). Previous research in insects and other arthropods shows that GPA2/GPB5 is typically expressed in the central nervous system, while LGR1 is distributed in peripheral tissues, including epithelia involved in osmoregulation, digestive tissues, and reproductive organs (35, 76, 77). Functional studies in *R. prolixus*, *A. aegypti*, and *Macrobrachium rosenbergii* have demonstrated roles for this pathway in developmental timing, ion and water balance, and the regulation of vitellogenesis and oogenesis (35, 76, 78). More specifically, in *D. melanogaster*, LGR1 is expressed in hindgut, Malpighian tubules, and other water-transporting epithelia, and its knockdown disrupts metamorphosis, reduces ecdysteroid titers, and decreases dehydration tolerance (76, 77). In *A. aegypti*, GPA2/GPB5 is expressed in neural tissue, and LGR1 is found at high levels in Malpighian tubules and hindgut, where this axis regulates ion transport and osmotic balance, inhibiting natriuresis and promoting kaliuresis during blood meal processing (35). Finally, in the crustacean *M. rosenbergii*, tandem knockdown of GPA2/GPB5 reduces vitellogenin expression in the hepatopancreas, while LGR1 knockdown increases vitellogenin receptor expression and oocyte size, indicating a role as a gonad-inhibiting factor (78). Together, these studies indicate that GPA2/GPB5-LGR1 signaling contributes to developmental progression, salt and water balance, and reproductive regulation in multiple invertebrate lineages.

The expression patterns we observed for GPA2 and LGR1 in *L. decemlineata* match several results reported in other invertebrates but also show species-specific differences. GPA2 was strongly expressed in the larval and adult CNS and across pupal stages, consistent with studies in *A. aegypti*, *R. prolixus*, and other arthropods where GPA2/GPB5 transcripts are concentrated in neural tissues (35, 76). LGR1 in our study was expressed primarily in the larval and adult midgut and in early larval instars, rather than in hindgut or Malpighian tubules as reported for *A. aegypti* and *D. melanogaster* (35, 76), however these tissues are closely linked both physically and functionally, so we did not consider this an anomaly. Functionally, our RNAi results showed that GPA2 and LGR1 knockdown influenced developmental transitions but had minimal effects on larval weight gain or survival. This partially aligns with findings in *D. melanogaster*, where LGR1 knockdown disrupts metamorphosis and alters ecdysteroid titers, or the hormone concentrations necessary for proper molting (79), and with reports in *M. rosenbergii* showing GPA2/GPB5 involvement in timing of reproductive development (78). However, we did not detect strong effects on osmoregulation, feeding, or reproduction that have been documented in other taxa, which may be related to the larval stadia evaluated in this experiment. Instead, the clearest response to knockdown in *L. decemlineata* was altered progression through pupal or late-larval stages, indicating that the GPA2/GPB5-LGR1 ligand-receptor pathway may contribute more narrowly to developmental timing in this species.

Specific to our study, across both injection and feeding assays, the semi-Markov models showed that time in state was a major determinant of developmental outcomes. Mortality was consistently highest immediately after a life stage transition and declined as individuals remained longer in that state, indicating that much of the baseline risk in development is tied to early-stage physiological vulnerability rather than treatment. Within this temporal framework, knockdown effects became clearer, particularly in several GPA2 and LGR1 feeding treatments producing consistent shifts in developmental transitions. This occurred most often during the larval to pupal and pupal to adult stages, where progression was either accelerated or delayed depending on dose and target gene. These results suggest that GPA2/LGR1 signaling influences the timing of developmental progression, rather than growth rate or survival overall.

### 4.4 Developmental dynamics, time-in-state effects, and methodological considerations

A central goal of the analysis was to disentangle treatment-specific effects on development from the underlying temporal dynamics of larval and pupal stages. Semi-Markov models were well suited to this problem because they incorporate time in state, allowing us to estimate how risk changes as individuals remain within a life stage. This approach helped clarify which observed mortality hazards were inherent to stage transitions and which were linked to GPA2 or LGR1 knockdown. Methodological considerations surrounding dsRNA delivery were also important. Injection produced broad, non-specific physiological costs, making treatment effects difficult to isolate in some contexts. However, the developmental patterns detected under injection were also present in the feeding assays, where baseline physiological stress was lower. This agreement across delivery methods supports the interpretation that the observed developmental shifts reflect true effects of GPA2 and LGR1 knockdown rather than any confounding effect from the dsRNA delivery method.

### 4.5 Broader implications and future directions in *L. Decemlineata* neuropeptide biology

Together, our findings on *L. decemlineata* underscore that neuroendocrine complexity in insects is far greater than previously appreciated. Although this study has limitations, most notable the lack of phenotypic data for every neuropeptide examined by qPCR due to experimental constraints, and no data for the GPB5 heterodimer, the statistical framework and phenotypic results for GPA2 and LGR1 offer a template that future studies can build on to further validate our conclusions. Additionally, uncovering neuropeptide network complexity goes beyond single pathway functional studies and individual transcript expression. Along with functional studies, *in vivo* sensors and signaling maps are two important methods moving forward to detangle and accurately report on neuropeptide signaling cascades (2). From a broader survey of tissue- and stage-specific expression patterns in *L. decemlineata* (41), several additional neuropeptides stand out to us as strong candidates for future functional work (Agatoxin-like 1, Allatostatin B, Allatostatin CCC, Calcitonin, Insulin-like peptide 2, Neuroparsin, and Orcokinin B). Each of these neuropeptides shows elevated expression across *L. decemlineata* tissues and life stages (**Supplementary Figure 1**; **Supplementary Excel File**) and have yet to be experimentally explored in this species.

Importantly, identifying these targets provides an exciting new avenue for how neuropeptide systems contribute to beetle development, metabolism, and environmental responses. As *L. decemlineata* continues to serve both as an emerging genetic model and a notorious agricultural pest, expanding functional characterization beyond GPA2 and LGR1 will be essential for understanding its endocrine system and may reveal undiscovered gene-targets for manipulating physiological pathways that are currently unexplored.

## Data Availability

The datasets presented in this study can be found in online repositories. The names of the repository/repositories and accession numbers can be found in the article/**Supplementary Material**.
