# Supplementary material for: Expression and RNAi characterization of neuropeptides underlying developmental regulation in the Colorado potato beetle
Source: Front Insect Sci. 2026 Aug 3;6:1862257. doi: 10.3389/finsc.2026.1862257 (PMC13478068; doi:10.3389/finsc.2026.1862257)
Supplement: Supplementary file 1 [file Supplementaryfile1.docx]

**Supplementary Table 1. Primer list for housekeeping genes used for qPCR**

Primer list for housekeeping genes used as controls in qPCR assays. The table includes candidate gene IDs, primer sequences, amplicon lengths, and brief functional descriptions.

| **Gene Description** | **Candidate Gene ID** | **Forward Primer Sequence** | **Reverse Primer Sequence** | **Notes** |
| --- | --- | --- | --- | --- |
| **ACTb** | LOC111512535 | 5’-CACCGTCCTCTCTGGACTGTTGGAAGGTGGACAGGGGGTA -3’ | 5’-CTGTTGGAAGGTGGACAGGG -3’ | Product length = 175  Actin-3, muscle-specific (B actin) |
| **RP4** | LOC111509998 | 5’-GGACCGAATCTGCCTTCCAA -3’ | 5’-ACCTTCTTCTGTGGAGCACG -3’ | Product length = 172  60S ribosomal protein L4 |
| **RP18** | LOC111508346 | 5’-TGATGGGTGCAACGTATACTCA -3’ | 5’-TAACCGTCGCTGGCCTTCAA -3’ | Product length = 154  Pre-mRNA processing factor 18 |

**Supplementary Table 2. Primer list for dsRNA and qPCR knockdown validation**

Primer sequences used for dsRNA synthesis and qPCR validation of neuropeptide and receptor knockdown in *Leptinotarsa decemlineata*. For each target gene, forward and reverse primer sequences, NCBI Gene/Protein IDs, expected product length, and design notes (exon coverage, non-overlapping 5′ regions for dsRNA specificity) are provided. Primer efficiency was determined by standard curve analysis using serial dilutions of cDNA template. Linear regression analysis of log(concentration) vs. Ct values yielded R² values ≥0.975 and amplification efficiencies between 94.5-100.3%.

| **Neuropeptide/Receptor** | **Protein and mRNA accession** | **Forward Primer Sequence** | **Reverse Primer Sequence** | **Primer type and product (bp)** | **R^2^** | **Slope** | **Efficiency (%)** |
| --- | --- | --- | --- | --- | --- | --- | --- |
| **LGR1** | XP_023016362.1,  XM_023160594.1 | 5’-TGAGCAGTTTGAGGCTACTTGATT -3’ | 5’-ACAGCAGTGAAAGGAATGGGT -3’ | Product length = 179  Covering exons, **flanking** | 0.985 | -3.41 | 95.2 |
| **LGR1** | XP_023016362.1 | 5’-AGAACGAATACCAGCCAATTCT -3’ | 5’-TGCAGTTTACGATTTGAGTGCAT -3’ | Product length = 141  LGR1 **5’end** (no overlap) | 0.985 | -3.41 | 95.2 |
| **GPA2** | XP_023027110.1, XM_023171342.1 | 5’-CATCCCGAACTGTGTCGAGT -3’ | 5’-GCAGGAACAAGTGACTGCTG -3’ | Product length = 220  GPA2 **flanking** | 0.982 | -3.35 | 96.8 |
| **GPA2** | XP_023027110.1 | 5’-GGATCAGCAAATGCGGGTGA -3 | 5’-AGCGTTCGTCGTCATGTGAA -3’ | Product length = 111  GPA2 **5’end** (very slight overlap) | 0.982 | -3.35 | 96.8 |
| **ITPA** | XP_023016721.1,  XM_023160953.1 | 5’-GTCGTCACCACCTAGCCAAA -3’ | 5’-GACCTAAGCCTTTGCCCCTT -3’ | Product length = 260  ITPA **flanking** (only on 5’ end) | 0.980 | -3.30 | 98.1 |
| **ITPA** | XP_023016721.1 | 5’-TGTGCGGGTTTCAGATGAGT -3’ | 5’-TGGTGATCTGTTCGCAGGAC -3’ | Product length = 125  ITPA **5’end** (no overlap) | 0.980 | -3.30 | 98.1 |
| **Tachykinin** | XP_023020111.1,  XM_023164343.1 | 5’-CCTTACGGATTTGTCGCGGT -3’ | 5’-CAGTACTAGCGCCATCCCAT -3’ | Product length = 196  Uncharacterized protein LOC111508740 | 0.988 | -3.44 | 94.5 |
| **Proctolin** | GEEF01088300.1,  XR_002721717.1 | 5’-TTCGCGGTCCTTTTGCTAGA -3’ | 5’-TGCTTTTGGTGTAGGGGCTT-3’ | Product length = 201  Uncharacterized LOC111502351 | 0.975 | -3.24 | 100.3 |
| **Baratin** | XP_023017557.1,  XM_023161789.1 | 5’-GCCAAATCTCGACCTGGACA -3’ | 5’-TTATTCCTGCTGTCTCCGCC -3’ | Product length = 198  Uncharacterized protein LOC111506660 | 0.983 | -3.37 | 96.2 |

**Supplementary Table 3. Relative Expression Values for Life Stage Specific qPCR data**

Relativized expression values for target neuropeptide and receptor genes (Proctolin, ITP-A, Baratin, Tachykinin, GPA2, and LGR1) across larval and pupal samples of *L. decemlineata.* Values represent normalized expression (used for downstream life stage-specific comparisons.

| Sample Name | Proctolin | ITP-A | Baratin | Tachykinin | GPA2 | LGR1 |
| --- | --- | --- | --- | --- | --- | --- |
| P4(M) | 0.14517387 | 1.14101598 | 0.34695778 | 0.56522443 | 0.48693938 | 0.31375639 |
| P5(M) | 4.9918E-05 | 3.26558251 | 0.36734888 | 0.49617425 | 0.53298445 | 0.17055628 |
| P7(M) | 0.01666055 | 4.24058278 | 0.47202673 | 0.49804112 | 0.7624684 | 0.00017147 |
| P6(F) | 0.56291567 | 2.99747557 | 1.74451083 | 4.21790414 | 1.66480789 | 0.00036944 |
| P8(F) | 0.33690231 | 0.82244555 | 1.12518313 | 0.71218811 | 0.58367558 | 0.78730974 |
| P9(F) | 6.14764416 | 0.3479214 | 0.5939627 | 0.38811876 | 1.1998319 | 2.08188646 |
| L3-11 | 0.21705807 | 0.87943695 | 0.05676367 | 0.03336487 | 0.01951292 | 0.4215039 |
| L3-12 | 0.82133177 | 0.80087809 | 0.05421656 | 0.05375492 | 0.0222927 | 2.5830445 |
| L3-13 | 0.24735043 | 0.61782058 | 0.1712306 | 0.11611865 | 0.01749567 | 1.19489337 |
| L1- 4 | 1.34734394 | 1.83718753 | 0.28146444 | 0.11094033 | 0.13432011 | 6.84236007 |
| L1-5 | 1.92712084 | 2.8361132 | 0.41590679 | 0.24579404 | 0.16384507 | 1.18981286 |
| L1-6 | 1.94997596 | 1.10343813 | 0.43832674 | 0.17526172 | 0.14575198 | 11.1256642 |

**Supplementary Table 4. Relative Expression Values for Tissue Specific qPCR data**

Relativized expression values for target neuropeptide and receptor genes (Proctolin, ITP-A, Baratin, Tachykinin, GPA2, and LGR1) across CNS, adipose, and midgut samples of *L. decemlineata.* Values represent normalized expression used for downstream tissue-specific comparisons.

| Sample Name | Proctolin | ITP-A | Baratin | Tachykinin | GPA2 | LGR1 |
| --- | --- | --- | --- | --- | --- | --- |
| ACNS3 | 119.4789 | 4.421499 | 15.927 | 4.900909 | 0.121051 | 0.206139 |
| ACNS2 | 11.6204 | 0.187346 | 0.102297 | 0.220497 | 41.16054 | 14.19384 |
| ACNS1 | 0.001744 | 2.922933 | 1.48606 | 2.24055 | 0.48594 | 0.82751 |
| AAD3 | 0.014786 | 1.054766 | 2.288159 | 6.27186 | 0.027668 | 0.047116 |
| AAD2 | 3.71E-05 | 0.434986 | 1.463059 | 4.137156 | 0.294585 | 0.017585 |
| AAD1 | 0.016098 | 2.934754 | 0.432774 | 3.465953 | 0.396774 | 0.018064 |
| AMG4 | 0.002708 | 0.327685 | 0.000349 | 0.588327 | 0.008004 | 0.002702 |
| AMG3 | 0.000497 | 0.267693 | 3.657719 | 8.856338 | 0.026636 | 0.001648 |
| AMG2 | 0.000976 | 0.585046 | 0.782649 | 6.521626 | 0.03509 | 6.938417 |
| AMG1 | 0.000484 | 0.248922 | 1.36376 | 0.660363 | 0.224208 | 10.11523 |
| LCNS3 | 81.45863 | 13.73844 | 43.70243 | 0.027059 | 1.938593 | 63.59008 |
| LCNS2 | 115.8048 | 37.09456 | 77.31982 | 0.482413 | 29.17885 | 28.54134 |
| LCNS1 | 87.32626 | 13.06214 | 44.67206 | 0.48118 | 67.96606 | 0.559048 |
| LAD3 | 3.83E-05 | 0.263672 | 0.10327 | 0.109808 | 0.630733 | 2.308409 |
| LAD2 | 7.18E-05 | 0.680112 | 1.060626 | 0.546077 | 0.410937 | 0.034051 |
| LAD1 | 0.000104 | 0.488236 | 0.000354 | 0.064975 | 1.127013 | 0.049161 |
| LMG3 | 0.001124 | 0.285011 | 0.736193 | 0.145249 | 0.003613 | 154.1207 |
| LMG2 | 0.001067 | 1.233425 | 1.036745 | 0.238576 | 0.087784 | 119.3132 |
| LMG1 | 4.57E-05 | 2.464422 | 0.951152 | 0.260434 | 0.021873 | 89.17075 |

**Supplementary Figure 1. Neuropeptide and LGR1 Expression Across *L. decemlineata* Tissues and Developmental Stages**

[Excel data sheet with Veenstra 2019 *Leptinotarsa* neuropeptides](https://docs.google.com/spreadsheets/d/1gU_lQDWiYLOoNBjLLhK_b6PGwxXUAAPf/edit?usp=sharing&ouid=111898079194219287860&rtpof=true&sd=true)

**Supplementary Figure 1.** Heatmap of log-transformed TPM values for all neuropeptides and GPCR receptors annotated in *L. decemlineata* identified from Veenstra (2019) using the CPB Gene Expression Atlas (Wilhelm & Xu 2025). Warmer colors indicate higher relative expression. Columns represent individual neuropeptide or receptor genes, and rows correspond to specific tissues and life stages. These atlas data were used to confirm gene annotations and validate tissue-specific expression patterns prior to qPCR analysis. Data consolidated for the heatmap produced here can be found in attached excel document: “Excel data sheet with Veenstra 2019 *Leptinotarsa* neuropeptides”

**Injection Materials and Methods, Results**

**dsRNA Injection Trial Methods**

**Injection of dsRNA**

Adult *L. decemlineata* were collected from the Hancock Agricultural Research Station (HARS) in Hancock, WI, and offspring were raised in the lab for the dsRNA injection trial. All injections were delivered to fourth instar larvae (L4) via microcapillary tube needles at a volume of 1 μl on the left side of the larvae between the 4^th^ and 5^th^ abdominal spiracles. Sample sizes for the injection assays were *n* = 50 per treatment. All injected larvae were transferred to 150 mm petri dishes with dampened filter paper in groups of 10 with fresh potato leaves for the duration of the experiment. Treatment groups for this trial included: C1 (uninjected control), C2 (1 μl H2O injected control), C3 (500 ng/μl control dsRNA), GD1 (500 ng/μl GPA2 dsRNA), GD2 (1000 ng/μl GPA2 dsRNA), LD1 (500 ng/μl LGR1 dsRNA), and LD2 (1000 ng/μl LGR1 dsRNA). Each treatment was replicated with 50 larvae per instar, for n = 350 total larvae. Control dsRNA used was the control template from the MEGAscript RNAi Kit (Thermo Fisher). Gene knockdown for both candidates in the L4 instar was confirmed via qPCR and gel electrophoresis.

**Phenotypic Assessments**

The gene knockdown trials were conducted over 28 days to allow development from larval stages through pupation into adulthood. All *L. decemlineata* were maintained in a growth chamber with 24 °C, 70% humidity and a photoperiod of 16 h light and 8 h dark per day. Phenotypic data was collected daily depending on life stage. Across the entire experiment, larvae were scored as alive, dead, or incapacitated, with incapacitated being defined as the inability to right oneself after being flipped dorsally. Life stage and time to development were also documented with each molt. For the injection trial, CPB life stages were injected following eclosion to the fourth instar, marked as pupating once fully molted atop shallow soil, and adult when pupal casing was molted.

**dsRNA Injection Trial Results**

**Larval Weight - Injection Trial**

Larval weight in the injection trial increased significantly over the assay period, with the GAM showing a positive temporal trend (*p* < 0.001), explaining 72.2% of the deviance (adj. R² = 67%) (**Figure S2**). A linear mixed model further confirmed a significant daily weight gain across all treatments (+0.0147 g/day, *p* < 0.001). No treatment was significantly different for initial larval weight (assay day 0; all *p* > 0.05). Among treatments, only the control dsRNA group showed a marginal trend toward faster growth compared to the uninjected control (+0.0090 g/day vs. controls, *p* = 0.057). Random intercept variance among dish replicates was minimal (σ² = 7.3 × 10⁻⁵) compared to residual variance (σ² = 1.1 × 10⁻³), indicating consistent treatment-level responses across replicates.

**Supplementary Figure 2. GAM fit: Injection Trial Weight Over Time.** Average larval weight (g) is shown across larval feeding assay days (1–7) for uninjected control (Control_Uninjected), water-injected control (Control_H₂O), dsRNA control (Control_dsRNA), and dsRNA treatments targeting GPA2 and LGR1 at low and high concentrations (GPA2_Low, GPA2_High, LGR1_Low, LGR1_High). Lines represent GAM-predicted fits with shaded ribbons indicating 95% confidence intervals.

**Rate of Defoliation - Injection Trial**

Patterns of potato leaf defoliation revealed temporal and treatment-related differences in feeding behavior following dsRNA exposure. The rate of defoliation (%) across assay days following injection was analyzed using beta regression with a logit link (pseudo-R² = 0.435; ϕ = 2.57, *p* < 0.001). On assay day 0, uninjected controls exhibited the highest percent defoliation (~95-100%), while all treatment groups were significantly lower (*x* < 0.001 for all treatment contrasts). Defoliation in uninjected controls declined significantly over time (*p* < 0.001). All other control and dsRNA treatments declined more rapidly, with significant Assay Day × Treatment interactions: Control H₂O (*p* < 0.001), Control dsRNA (*p* < 0.001), GPA2 Low (*p* < 0.001), GPA2 High (*p* < 0.001), LGR1 Low (*p* < 0.001), and LGR1 High (*p* < 0.001). The number of surviving beetles per dish was positively associated with defoliation (*p* = 0.008). By assay days 6-7, all treatment groups converged to ~5-15% defoliation, while uninjected controls remained marginally higher (~15-20%) (**Figure S3**).

**Supplementary Figure 3. Beta-regression: Injection Trial Defoliation.** Defoliation over time by treatment group, measured in percent of the potato leaf defoliated. Lines represent predicted values from beta regression; shaded ribbons denote standard errors.

## **Survival Analysis - Injection Trial**

Kaplan-Meier curves differed among treatments (log-rank χ² = 44.0, df = 6, *p* = 8×10⁻⁸), but there were no significant differences in survival among injected treatments (*padj* > 0.09). Uninjected control showed higher survival than every injected group (pairwise log-rank, Bonferroni-adjusted): control H₂O *p* = 4.9×10⁻⁵; control dsRNA *p* = 5.3×10⁻⁵; GPA2_Low *p* = 3.0×10⁻⁹; GPA2_High *p* = 0.042; LGR1_Low *p* = 0.0014; LGR1_High *p* = 1.6×10⁻⁷.

**Supplementary Figure 4. Injection Trial Kaplan-Meier Survival by Treatment.** Survival probability of *L. decemlineata* larvae across assay days (0-28) by treatment group. Shaded areas represent 95% confidence intervals, and numbers below indicate individuals at risk at each time point.

## **Development - Injection Trial**

For the life stage transition between larva and pupa, there were significant differences between the control and treatment groups. (1→3; **Table S5**). Relative to the uninjected control (C1), transition hazards were significantly reduced in the H_2_O injected control group (C2; coef -1.505, HR = 0.222, *p* = 0.0073), dsRNA injected control group (C3; -1.499, HR = 0.223, *p* = 0.017), low dose GPA2 dsRNA treatment (GD1; -2.833, HR = 0.0588, *p* = 0.0060), high dose GPA2 dsRNA treatment (GD2; -1.053, HR = 0.349, *p* = 0.028), and high dose LGR1 dsRNA treatment (LD2; -1.766, HR = 0.171, *p* = 0.0188); low dose LGR1 dsRNA treatment (LD1) was not significant. The overall model was significant (likelihood-ratio test [LRT] = 22.45 on 6 df, *p* = 0.0010; n = 350, events = 39). Overall, larval progression to pupation was slower in the injected control and dsRNA treatments than in the uninjected control, indicating treatment-associated and injection-induced delays in development.

**Supplementary Table 5. Model 1→3: Larvae to Pupae**

|  | Coefficient (β) | Hazard Ratio | SE (β) | z-statistic | p-value |
| --- | --- | --- | --- | --- | --- |
| Treatment C2 | -1.50507 | 0.22200 | 0.56055 | -2.685 | 0.00725 |
| Treatment C3 | -1.49872 | 0.22342 | 0.63016 | -2.378 | 0.01739 |
| Treatment GD1 | -2.83308 | 0.05883 | 1.03187 | -2.746 | 0.00604 |
| Treatment GD2 | -1.05321 | 0.34882 | 0.48033 | -2.193 | 0.02833 |
| Treatment LD1 | -0.75192 | 0.47146 | 0.45455 | -1.654 | 0.09809 |
| Treatment LD2 | -1.76607 | 0.17100 | 0.75135 | -2.351 | 0.01875 |

Likelihood ratio test=22.45 on 6 df, ***p*=0.001003**

n= 350, number of events= 39

For the life stage transition between beetles deemed incapacitated to death, there were no significant differences between the control and treatment groups, but a significant effect of time in state (2→5; **Table S6**). The semi-Markov specification indicated a decreasing hazard with time in state ($B(t)$; coef -1.471, HR/day = 0.230, *p* = 0.071), or higher chance of survival the longer organisms survived past life stage transition. Treatment effects were not significant (all *p* ≥ 0.71); however, the overall model was significant (LRT = 49.71 on 7 df, *p* = 1.65 × 10⁻⁸; n = 52, events = 24). The highest risk of death occurred soon after incapacitation and declined with each additional day spent in that state, with no clear treatment differences once incapacitated.

**Supplementary Table 6. Model 2→5:** Incapacitated to Dead

|  | Coefficient (β) | Hazard Ratio | SE (β) | z-statistic | p-value |
| --- | --- | --- | --- | --- | --- |
| $B(t)$ | -1.47097 | 0.22970 | 0.81485 | -1.805 | 0.071 |
| Treatment C2 | 0.07095 | 1.07353 | 0.76799 | 0.092 | 0.926 |
| Treatment C3 | 0.32016 | 1.37735 | 0.86817 | 0.369 | 0.712 |
| Treatment GD1 | 0.07095 | 1.07353 | 0.91641 | 0.077 | 0.938 |
| Treatment GD2 | 0.32016 | 1.37735 | 1.22626 | 0.261 | 0.794 |
| Treatment LD1 | 0.30684 | 1.35913 | 0.84630 | 0.363 | 0.717 |
| Treatment LD2 | 0.20095 | 1.22256 | 0.78454 | 0.256 | 0.798 |

Likelihood ratio test=49.71 on 7 df, ***p*=1.645e-08**

n= 52, number of events= 24

For the life stage transition between pupa to adult, there were significant differences between the control and treatment groups (3→4; **Table S7**). A higher progression rate was detected for LD2 (coef 2.197, HR = 8.995, p = 0.0115). C3 showed a trend towards a higher progression rate (1.394, HR = 4.029, p = 0.0545) and GD2 a trend towards a lower progression rate (-1.122, HR = 0.326, p = 0.0745). Time-in-state terms were not significant ($B(t)$: 1.072, HR/day = 2.920, p = 0.802; $I(Bt^{2})$: -0.122, HR = 0.885, p = 0.692), however the overall model was significant (LRT = 24.48 on 8 df, p = 0.0019; n = 39, events = 33). Progression from pupa to adult was substantially faster in LD2 relative to the uninjected control, while time spent in the pupal state itself did not show an independent effect on the transition rate.

**Supplementary Table 7. Model 3→4:** Pupae to Adult

|  | Coefficient (β) | Hazard Ratio | SE (β) | z-statistic | p-value |
| --- | --- | --- | --- | --- | --- |
| $B(t)$ | 1.0715 | 2.9197 | 4.2818 | 0.250 | 0.8024 |
| $(Bt^{2})$ | -0.1220 | 0.8852 | 0.3076 | -0.397 | 0.6917 |
| Treatment C2 | 0.4876 | 1.6285 | 0.5987 | 0.815 | 0.4153 |
| Treatment C3 | 1.3935 | 4.0290 | 0.7248 | 1.923 | 0.0545 |
| Treatment GD1 | 0.1911 | 1.2106 | 1.0832 | 0.176 | 0.8600 |
| Treatment GD2 | -1.1215 | 0.3258 | 0.6289 | -1.783 | 0.0745 |
| Treatment LD1 | 1.1395 | 3.1253 | 0.7684 | 1.483 | 0.1381 |
| Treatment LD2 | 2.1967 | 8.9953 | 0.8697 | 2.526 | 0.0115 |

Likelihood ratio test=24.48 on 8 df, ***p*=0.001905**

n= 39, number of events= 33

Models 3→2 (pupae to incapacitated) and 4→2 (adults to incapacitated) had 0 events, so model results for both are NA. Models 3→5 (pupae to dead) and 4→5 (adult to dead) had very few events, 5 and 7 events, respectively, so there is minimal statistical inference able to be made.

**Supplementary Figure 5. RNAi knockdown efficiency of GPA2 and LGR11 following feeding and injection treatments.** Relative expression levels of GPA2 and LGR1 following RNAi treatment delivered by feeding or injection at 24 h post-treatment on whole body larvae. Gene expression was quantified by qPCR and normalized using the 2^^-ΔΔCt^ method relative to control groups (dose = 0). Bars represent mean ± SEM for each dsRNA dose tested. Statistical comparisons between treatment and control groups were performed using one-way ANOVA followed by post hoc testing. Reduced expression levels indicate successful RNAi-mediated knockdown of target genes.
